# The estimated cost of dementia in Japan, the most aged society in the world

**DOI:** 10.1371/journal.pone.0206508

**Published:** 2018-11-12

**Authors:** Mitsuhiro Sado, Akira Ninomiya, Ryo Shikimoto, Baku Ikeda, Toshiaki Baba, Kimio Yoshimura, Masaru Mimura

**Affiliations:** 1 Department of Neuropsychiatry, Keio University School of Medicine, Tokyo, Japan; 2 Center for Stress Research, Keio University, Tokyo, Japan; 3 Internal medicine Division, Ikeda hospital, Nonoichi, Japan; 4 National Institute of Mental Health, National Center of Neurology and Psychiatry, Tokyo, Japan; 5 Department of Health Policy and Management, Keio University School of Medicine, Tokyo, Japan; University of West London, UNITED KINGDOM

## Abstract

**Objective:**

Dementia has become a global critical issue. It is estimated that the global cost of dementia was 818 billion USD in 2015. The situation in Japan, which is the most aged country in the world, should be critical. However, the societal cost of dementia in Japan has not yet been estimated. This study was designed to estimate cost of dementia from societal perspective.

**Design:**

We estimated the cost from societal perspective with prevalence based approach.

**Setting, participants and measures:**

Main data sources for the parameters to estimate the costs are the National Data Base, a nationwide representative individual-level database for healthcare utilization, the Survey of Long-Term Care Benefit Expenditures, a nationwide survey based on individual-level secondary data for formal long-term care utilization, and the results of an informal care time survey for informal care cost. We conducted the analyses with ‘probabilistic modeling’ using the parameters obtained to estimate the costs of dementia. We also projected future costs.

**Results:**

The societal costs of dementia in Japan in 2014 were estimated at JPY 14.5 trillion (se 66.0 billion). Of these, the costs for healthcare, long-term care, and informal care are JPY 1.91 trillion (se 4.91 billion), JPY 6.44 trillion (se 63.2 billion), and JPY 6.16 trillion (se 12.5 billion) respectively. The cost per person with dementia appeared to be JPY5.95 million (se 27 thousand). The total costs would reach JPY 24.3 trillion by 2060, which is 1.6 times higher than that in 2014.

**Conclusions:**

The societal cost of dementia in Japan appeared to be considerable. Interventions to mitigate this impact should be considered.

## Background

Dementia has become a global critical issue, the situation of which could be described as almost ‘under pandemic’. The number of people with dementia across the world is estimated 46.8 million in 2015 and will rise to 131.5 million by 2050 across the world [1]. The impairment caused by the disease ‘characterized by multiple cognitive defects’ [2] is extensive in wide range, including general intelligence, learning and memory, language, problem solving, orientation, perception, attention and concentration, judgment, and social abilities [3]. Given such clinical features, the effects of dementia extend to people’s families and wider society. Both a rapid expansion in the number of people living with dementia and its clinical features contributes to increasing the societal burden. It is estimated that the global cost of dementia was 818 billion USD in 2015 and will reach 2 trillion USD in 2030 [1]. The magnitude of the costs caused by the disorder is attracting huge attention especially in the developed countries. The societal costs of dementia in the US have been estimated to be 157 to 215 billion US dollars in US in 2010 [4], 26 billion pounds in UK in 2013 [5], and 177 billion Euro in whole of Europe and regions according to the classification by United Nations in 2008 [6]. These estimates were influential in the development of policies on dementia at the national or regional level [7][8][9].

The situation in Japan is even more critical. The reason is that Japan is the most aged country in the world [10], where already more than 4.6 million people with dementia live [11]. According to calculation by the authors based on published data, the worldwide prevalence of dementia per capita was around 0.6% in 2015, and is projected to be 1.4% in 2050. In other developed countries such as the UK, the prevalence per capita was already 1.5% in 2013 and still remains approximately 3% in 2050. In contrast, in Japan the prevalence was already beyond 3% in 2015 and will reach close to 9% of the population in 2050 [5][12][13]. These figures illustrate the impact of dementia in Japan. In order to address this issue, the government of Japan launched and updated a national dementia plan in 2012 [14] and in 2015 [15]. However, the plan did not consider the societal costs of dementia, which would bring useful information when evaluating the impact of this disease and help address questions concerning resource allocation. Therefore this study was designed to estimate societal cost of dementia in Japan that will provide evidence based information regarding management of dementia and will be helpful for policy maker to allocate resources for this purpose.

## Method

### Design

We estimated the annual costs from a societal perspective, considering the cost implications of use of healthcare, formal long-term care (LTC) services and also the costs of the care provided by informal (mostly family) careers, which constitute an important part of the costs of dementia [16][17]. This research was approved by the Ethical committee at Keio University School of Medicine.

### Process of estimation

First we constructed the formulae to estimate the cost of healthcare, LTC and informal care respectively (Table 1). The formulae for estimating each component of the cost were developed considering the methodologies adopted in previous studies [1, 18–21] and the data available under the Japanese setting. Next, we obtained data related to the parameters in the formulae above based on the individual and national level data. Then, we conducted the cost estimates by imputing the parameters’ value into the formulae in Table 1. However, each parameter bore an uncertain mean value. Therefore, the analyses of cost estimates were conducted through probabilistic modeling in order to reflect the uncertainty in the input parameters [22]. In probabilistic modeling, the value of each parameter was determined randomly in accordance with the mean and its distribution pattern. The textbook should be referred to for details [22]. Finally, we evaluated the future projection of the cost on the basis of forecast of the demographic data. We assumed that all people with dementia are 40 years or older.

**Table 1:** Formulae to estimate each component of the cost.

Health Care cost	*TC*_*hc*_ *= HC*_*in*_ *+ HC*_*out*_ $HC_{in\left(out\right)}=\sum_{j=1}^{10}N_{dem-in\left(out\right)-j}\times MC_{dem-in\left(out\right)-j}\times 12$
Long-Term Care (LTC) cost*^1^^,^*^2^	$TC_{ltc}=\sum_{s=1}^{2}\sum_{m=1}^{12}\sum_{i=1}^{7}N_{s-m-i}\times \;R_{dem-s-m-i}\times \;C_{ltc-s-m-i}\times \;RR_{dem-s}$
Informal care cost*^1^	$TC_{icc}=\sum_{i=1}^{7}N_{ltc-i}\times R_{dem-out-i}\times (T_{ic-adl-i}\times UC_{adl}+\times T_{ic-iadl-i}\times UC_{iadl})\times 52$

Abbreviation

<Healthcare cost>

TChc = total healthcare cost, HCin = inpatient cost, HCout = outpatient cost, Ndem-in(out)-j = the monthly number of the inpatients (outpatients) with dementia at sex and age band of j

MCdem-in(out)-j = monthly mean cost of inpatient (outpatient) at sex and age band of j

<LTC cost>

TCltc = total LTC cost of dementia, Ns-m-i = the number of the people with care needed level i at month of m receiving LTC services of s

Rdem-s-m-i = the rate of the people with dementia among all users with care needed level i at month of m receiving LTC services of s

Cltc-s-m-i = the average cost of LTC service of s with care needed level i of those without dementia at month of m

RRdem-s = the relative ratio of the average cost of LTC service of s for those with dementia against that of those without dementia

<Informal care cost>

TCicc = total informal care cost, Nltc-i = the number of people with care needed level i using home care services

Rdem-out-i = rate of the people with dementia among all users of home care services with care needed level i

Tic-adl-i = informal care time for ADL of the people with dementia with care needed level i using home care services (hours/ week)

UCadl = unit cost of informal care for ADL (JPY/hour)

Tic-iadl-i = informal care time for IADL of the people with dementia with care needed level i using home care services (hours/ week)

UCiadl = unit cost of informal care for IADL (JPY/hour)

*1 Care needed level ranging from 1 to 7 represents from support needed level 1 to nursing care level 5

*2 Home care services in LTC schema include three different services (i.e. home-based service, home-based support, and small-scale local service)

### The formulae and the process to obtain data parameters

#### Healthcare costs

The formulae to estimate healthcare cost are shown in Table 1. As indicated in the upper formula in the healthcare cost section in Table 1, we assumed that the total healthcare cost due to dementia was the sum of the inpatient and the outpatient costs. The lower formula in the same section indicated that the inpatient or outpatient costs were estimated by integrating inpatient or outpatient costs sorted according to sex and age band. There were five age bands (i.e. 40–49, 50–59, 60–69, 70–79, and 80-). Then, we aggregated ten segments (2 genders × 5 age bands) of inpatient or outpatient healthcare costs sorted by sex and age band (the variable ‘*j*’ in the lower formula represented ‘segment’). The inpatient or outpatient healthcare cost in each segment was calculated by multiplying the monthly number of inpatients or outpatients by the monthly mean inpatient or outpatient costs. To obtain data for the parameters in the formula (the monthly number of patients, and the monthly mean cost sorted according to sex and age band), we accessed a sampling data set (SDS) extracted from the National Data Base (NDB), a database of all national healthcare insurance receipts. The SDS, a dataset created and provided by the Ministry of Health, Labour and Welfare of Japan, is comprised of the randomly extracted 1% for outpatient, and 10% for inpatient receipts respectively from NDB for the one-month period of October 2011.

We first counted the monthly number of patients with a clinical diagnosis of dementia stratified by sex and age band. The number of patients was adjusted to reflect the population change from 2011 to 2014. Next, we conducted multivariate regression analyses using SDS to estimate outpatient and inpatient cost respectively. The healthcare cost was set to be the independent variable, while sex, age, each diagnostic, and the total number of days of use of healthcare services were entered into the model as dependent variables. Diagnoses including dementia were classified into 17 subgroups using the Charlson comorbidity index [23], and imputed into the model as dichotomous variable. The model and the results are shown in the S1 and S2 Files.

Once the model was constructed, the healthcare cost attributable to dementia per patient was predicted by entering sex, age, diagnosis, and the total number of days of use of healthcare services of each person with dementia into the model. In order to exclude the healthcare costs relating to comorbid diseases and to estimate the cost attributable specifically to dementia as accurately as possible, all diagnoses other than dementia were hypothesized not to exist by entering a zero into the dichotomous variables of diagnosis classified by the Charlson comorbidity index [23].

By summing up the predicted cost attributable to dementia for each person with dementia by age and sex, we obtained the mean and the standard error (SE) of the monthly healthcare cost stratified by sex and age.

#### Long term care costs

In Japan, LTC services are provided under LTC insurance schema. As shown in the formula in Table 1, LTC costs were basically calculated by multiplying the number of people receiving LTC services, the probability of people with dementia among all users receiving LTC services, the average cost of LTC services of those without dementia, and the relative ratio of the average cost of LTC services for those with dementia against that of those without dementia. The degree of care needed (represented as *i* in the formula) was divided into seven categories, that is, two “support needed” levels plus five “nursing care” levels, and the services provided under LTC insurance schema were categorized into home services and institutional services (*s* in the formula). Furthermore, the Survey of Long-Term Care Benefit Expenditures provided the respective monthly data related to the parameters shown in the formula. Therefore, total LTC costs were estimated by aggregating 168 segments of LTC costs (7 care needed level × 12 months × 2 categories of services).

To obtain the data of the parameters in the formula in Table 1, first, we accessed to individual-level secondary data provided from the Survey of Long-Term Care Benefit Expenditures, a nationwide survey [24][25] to obtain the variables, the number of users, the rate of people with dementia among all users and the average LTC costs, relevant to each care needed level. The average LTC costs reported in this survey [24][25] were the comprehensive average costs, which included both those of people with and without dementia. In order to estimate the average cost attributable specifically to dementia, we estimated the relative ratio of the cost among those with and without dementia inside the each care needed level, by conducting regression analysis using the individual receipts for service provision of the LTC insurance from a local municipality (n = 2,245). See the S1 File for the details about the process of estimating ‘relative ratio.’ By weighing the ‘relative ratio’, we estimated the average LTC costs of the people with dementia in each care needed level. Because the individual receipts of healthcare and LTC are administered separately, it was impossible to combine the data of medical condition (i.e. diagnosis) and service provision of LTC at individual level. Therefore, with respect to LTC cost, it was impossible to exclude the effect of comorbid diseases to the LTC cost.

#### Informal care costs

As mentioned earlier, dementia is associated with a progressive loss of cognitive and intellectual abilities such as memory, judgment, and abstract thinking. People with profound cognitive disability need assistance in almost every aspect of daily living [26]. Therefore, estimating the unpaid care costs incurred mostly from families (informal care cost) is crucial.

As indicated in the formula in Table 1, the basic strategy of estimating the informal care costs was integration of the informal care costs sorted according to care needed level (represented as ‘*i*’ in the formula). Then, the informal care cost at each care needed level was calculated by multiplication of the number of people with dementia at each care needed level using home services (represented as *N*_*ltc-i*_ *× R*_*dem-out-i*_ in the formula), the informal care cost per person with dementia (‘*T*_*ic-adl-i*_*× UC*_*adl*_ *+ ×T*_*ic-iadl-i*_*× UC*_*iadl*_’), and 52 weeks. Data for the parameters in the formula were obtained through the following processes.

As there was no available data on the amount of time providing informal care to people with dementia, we conducted a survey. We distributed questionnaires to caregivers via hospitals, clinics, nursing homes and caregiver support organizations in 38 out of the 47 prefectures in Japan. Participants the recorded time spent on providing informal care over a 1-week period. The details of the samples are summarized in the S2 File.

We developed a model that predicts time spent on informal care by conducting regression analysis using data from the survey. All independent variables were entered into a model with the forced entry method. Informal care time, the dependent variable in the model, included solely time spent providing support with Activities of Daily Living (ADL) and Instrumental Activities of Daily Living (IADL).

Because the sample of this survey was not a drawn from a random sample of the population, some characteristics of the sample members, such as age, sex, existence of a others living together, total amount of time providing care, comorbid diseases, and Behavioral and Psychological Symptoms of Dementia (BPSD) might not be representative. Therefore, in order to obtain an estimate the mean and SE of informal care provision time in Japan, we conducted ‘probabilistic re-sampling’ for 10,000 times by extrapolating nationwide representative data relevant to age, sex, and whether living alone or not, in accordance with the mean and distribution of each parameter, into the model. The details about the regression analyses and the probabilistic re-sampling are shown in the S1 and S2 File.

There is no clear consensus about how to apply an economic cost to informal care time [27]. In this study, a replacement cost approach was applied to the time spent providing ADL-type care, while an opportunity cost approach was applied to IADL-type care. We used the fee for ‘physical care’ in LTC services as the unit cost under the replacement approach. For the opportunity cost approach we used the expected mean lost wages among the samples calculated on the basis of the nationwide survey of the mean wage [28] and labour participation rate by sex and age [29]. Sensitivity analysis was also performed as follows.

Case 1: The opportunity cost approach was applied both to ADL and IADL.
Case 2: The replacement cost was applied both to ADL and IADL.
Case 3: The replacement cost was applied to ADL and the opportunity cost was applied to IADL and supervision time (SV).

Due to constraints of data availability, the scope of the estimate was informal care cost of the people with dementia who live at home and use LTC services.

### Estimating the total costs of dementia

Once we obtained all the costs parameters, we conducted ‘probabilistic re-sampling calculation’ for 1,000 times on the basis of the mean and the se of each parameter in the formulae above to estimate the health care services, long-term formal care and informal care, and for the total cost of dementia.

### Future projection

We also estimated how the societal costs of dementia will vary in future. Our estimate assumes that factors other than the change of the population by gender and age (i.e. factors such as incidence of dementia, engagement with healthcare services, use of formal care services, and informal care time, and unit cost for each care) remain constant. We assumed a discount rate of 3%. The change in cost is estimated as the change in the absolute cost and the cost per worker. The data of the projected number of the population by sex and age were obtained from the Population Projection for Japan: 2011–2060 [30].

Analyses were conducted by STATA ver. 13. and Excel 2011. The details of the process and the results of estimate not appeared in the manuscript are shown in S1 and S2 File. The costs in US Dollars is based on the averaged purchasing power parity at 2014 (i.e. USD 1 is equal to JPY 128.82) [31].

## Results

### Parameters

The parameters related to healthcare costs are listed in Table 2. Those relevant to LTC and informal care cost are in Table 3. The probability that LTC service users have dementia ranges between 0.193 and 0.974 depending on the care needed level. With respect to informal care time, 1,685 questionnaires out of 4,236 distributed were returned (response rate 40%). Of these, 1,482 were correctly completed and were included into the analysis. Following the regression analysis and the extrapolation of nationwide representative data into the model, the informal care time (hours/ week) on average (mean (SE)) were 24.97 (0.057), these varied by care needed level ranging between 10.19 (0.050) and 38.16 (0.062). The breakdown of the informal care time is as shown in Table 3.

**Table 2:** Parameters related to estimate healthcare cost.

	age	male					female				
		no. of the patients with dementia (per month)*		average cost per patient (JPY per month)			no. of the patients with dementia (per month)*		average cost per patient (JPY per month)		
		n	distribution	mean	se	distribution	n	distribution	mean	se	distribution
inpatient	40–49	299	determinisitic	378,267	17,891	gamma	159	determinisitic	345,103	24,938	gamma
	50–59	1,123	determinisitic	382,665	9,702	gamma	734	determinisitic	355,825	9,929	gamma
	60–69	6,633	determinisitic	397,253	4,393	gamma	5,112	determinisitic	363,544	4,451	gamma
	70–79	24,484	determinisitic	383,375	3,185	gamma	27,882	determinisitic	351,120	2,618	gamma
	80-	50,568	determinisitic	350,563	2,347	gamma	118,197	determinisitic	326,581	1,289	gamma
outpatient	40–49	1,067	determinisitic	41,277	5,283	gamma	638	determinisitic	35,066	4,967	gamma
	50–59	3,001	determinisitic	43,647	4,040	gamma	3,284	determinisitic	40,837	4,898	gamma
	60–69	27,866	determinisitic	39,471	1,113	gamma	32,516	determinisitic	40,872	1,456	gamma
	70–79	187,141	determinisitic	43,620	609	gamma	289,829	determinisitic	41,652	479	gamma
	80-	395,435	determinisitic	40,222	348	gamma	1,041,396	determinisitic	37,960	216	gamma

* the number of the patients represents those who consume healthcare service during one month.

**Table 3:** Parameters related to estimating LTC cost and informal care cost.

home care service*^1^	care needed level	monthly number of LTC service (home care) users(thousand)	probability of people with dementia*^2^		monthly number of the users with dementia (thousand)	average cost (JPY thousand)	Relative Ratio (RR) of LTC cost with the dementia against without dementia (ln(RR))*^3^						informal care time (hrs/week)*^4^							
							home-based services		home-based support		small-scale local services		ADL		IADL		ADL+IADL		SV	
			mean	se			ln(RR)	se	ln(RR)	se	ln(RR)	se	mean	se	mean	se	mean	se	mean	se
	1	480	0.193	0.004	93	30	0.339	0.046	0.331	0.017	0.031	0.162	2.39	0.032	7.80	0.023	10.19	0.050	13.50	0.066
	2	584	0.153	0.002	90	50	0.339	0.046	0.331	0.017	0.031	0.162	11.11	0.035	10.70	0.025	21.81	0.056	25.37	0.068
	3	840	0.459	0.002	386	103	0.339	0.046	0.331	0.017	0.031	0.162	6.55	0.033	12.37	0.024	18.92	0.053	22.96	0.068
	4	799	0.444	0.002	355	136	0.339	0.046	0.331	0.017	0.031	0.162	9.71	0.036	12.63	0.025	22.34	0.056	27.63	0.070
	5	494	0.554	0.002	273	206	0.339	0.046	0.331	0.017	0.031	0.162	14.37	0.038	12.45	0.026	26.82	0.059	28.19	0.073
	6	351	0.638	0.002	224	242	0.339	0.046	0.331	0.017	0.031	0.162	19.19	0.038	14.17	0.027	33.35	0.060	31.56	0.072
	7	240	0.799	0.002	192	287	0.339	0.046	0.331	0.017	0.031	0.162	23.66	0.039	14.51	0.027	38.16	0.062	31.35	0.071
	total	3,788	0.425	0.001	1,612	131							12.38	0.036	12.59	0.026	24.97	0.057	26.66	0.070
institutional care services*^5^	care needed level	monthly number of LTC service (institutional care) users(thousand)	probability of people with dementia*^2^		monthly number of the users with dementia (thousand)	average cost (JPY thousand)	Relative Ratio (RR) of LTC cost with the dementia against without dementia (ln(RR))*^3^													
							instituional care													
			mean	se			ln(RR)	se												
	1	n/a	n/a	n/a	n/a	n/a	n/a	n/a												
	2	n/a	n/a	n/a	n/a	n/a	n/a	n/a												
	3	52	0.731	0.002	38	245	0.016	0.075												
	4	108	0.798	0.001	86	261	0.016	0.075												
	5	194	0.892	0.001	173	279	0.016	0.075												
	6	280	0.937	0.000	263	301	0.016	0.075												
	7	274	0.974	0.000	267	325	0.016	0.075												
	total	908	0.910	0.000	826	296														

*1 home care services include home-based services, home-based support, small-scale local services.

*2 Beta distribution is assumed

*3 Lognormal distribution is assumed

*4 Gamma distribution is assumed

*5 Institutional care services are available only for those categorized in nursing care level (i.e. care needed level 3 and over)

Abbreviation: n/a: not applicable

### Cost of dementia

The total healthcare costs of dementia (mean (SE)) were estimated to be JPY 1.91 trillion (4.91 billion) (USD 14.8 billion (38 million)). Of these, inpatient costs were JPY 970 billion (2.79 billion) (USD 7.5 billion (22 million)), outpatient costs were JPY 941 billion (3.96 billion) (USD 7.3 billion (31 million)). With respect to LTC costs, they came to a total of JPY 6.44 trillion (63.2 billion) (USD 50.0 billion (491 million)). The costs of home care and institutional care were JPY 3.53 trillion (60.2 billion) (USD 27.4 billion (467 million)) and JPY 2.92 trillion (17.4 billion) (USD 22.6 billion (135 million)) respectively. The total informal care costs were estimated to be JPY 6.16 trillion (12.5 billion) (USD 47.8 billion (97 million)). In sensitivity analysis, informal care costs in case 1, 2, and 3 were JPY 2.02 trillion (4.0 billion) (USD 15.7 billion (31 million)), JPY 7.63 trillion (15.2 billion) (USD 59.2 billion (118 million)), and JPY 8.32 trillion (16.4 billion) (USD 64.6 billion (127 million)) respectively. By summating the estimates for all costs components, the societal costs of dementia in Japan in 2014 were estimated at JPY 14.5 trillion (66.0 billion) (USD 112.7 billion (513 million)). The details are shown in Table 4.

**Table 4:** Total cost and cost per person with dementia in 2014.

	total cost				cost per person with dementia **					
	JPY(million)		USD(million)*		JPY(thousand)			USD(thousand)*		
	mean	se	mean	se	mean		se	mean		se
healthcare cost										
total	1,911,459	4,905	14,838	38	784		2	6.1		0.02
outpatient	970,261	2,793	7,532	22	398		1	3.1		0.01
inpatient	941,198	3,962	7,306	31	386		2	3.0		0.01
LTC cost										
total	6,443,243	63,234	50,017	491	2,643		26	20.5		0.20
home care services	3,528,022	60,176	27,387	467	2,189	^$^	37	17.0	^$^	0.19
institutional services	2,915,221	17,390	22,630	135	3,528	^$ $^	21	27.4	^$ $^	0.06
informal care cost						^ ^			^ ^	
base case	6,159,280	12,538	47,813	97	3,822	^$^	8	29.7	^$^	0.04
sensitivity analysis 1	2,019,419	3,985	15,676	31	1,253	^$^	2	9.7	^$^	0.01
sensitivity analysis 2	7,631,149	15,162	59,239	118	4,735	^$^	9	36.8	^$^	0.05
sensitivity analysis 3	8,315,353	16,416	64,550	127	5,160	^$^	10	40.1	^$^	0.05
total cost of dementia	14,513,981	66,031	112,669	513	5,954		27	46.2		0.21

*USD 1 is equal to JPY 128.82 based on the purchasing power perity at June 2014 (http://www.iima.or.jp/research/ppp/index.html)

** the number of the people with dementia is assumed to be 2,437 thousand in total except figures with $, $ $.

$ the number of the people with dementia is assumed to be those at home (ie. 1,611 thousand)

$ $ the number of the people with dementia is assumed to be those at institution (ie. 826 thousand)

### Cost per person of dementia in Japan

If we assume the number of people with dementia in Japan is equal to the estimated number of people using LTC services who have dementia (i.e. 2.4 million), the cost per person with dementia (mean (SE)) is JPY5.95 million (27 thousand). Of this, the mean healthcare cost, LTC cost, and informal care cost per person per year would be JPY 784 thousand, JPY 2,643 thousand and JPY 3,822 thousand respectively.

### Future projection

The societal costs of dementia would reach JPY 24.3 trillion by 2060, which is 1.6 times higher than that in 2014. When we calculated cost per worker, it appeared to be 2.8 times higher than that at present (Table 5).

**Table 5:** Future projection of the cost of dementia.

year	2015	2020	2025	2030	2035	2040	2045	2050	2055	2060
total cost (billion JPY/year)	15,008	17,419	19,444	21,381	22,923	22,937	22,546	22,768	23,604	24,262
index*^1^	1.00	1.16	1.30	1.42	1.53	1.53	1.50	1.52	1.57	1.62
cost per labour force (thousand JPY/year)	195	237	274	316	361	396	421	455	502	549
index*^1^	1.00	1.21	1.40	1.62	1.85	2.03	2.16	2.33	2.57	2.81
labour force population (aged between 15–64) (thousand)*^2^	76,818	73,408	70,845	67,730	63,430	57,866	53,531	50,013	47,063	44,183

*1 fugure in 2015 is set as reference

*2 cited from National Institute of Population and Social Security Research. Population Projections for Japan. Tokyo: 2012.

## Discussion

Despite the rapidly increasing prevalence of dementia in Japan, this is the first study to estimate the societal costs of dementia in Japan.

### Main component of the total cost

We found that the societal cost due to dementia in Japan is JPY 14.5 trillion (USD 112 billion). Nearly 90% of the total cost is attributable to formal and informal care. And this result is in accordance with the results of previous studies which indicate that the dementia brings huge burden to the care givers irrespective of formally or informally [4, 5]. Especially it is surprising that informal care cost is almost equivalent to the formal LTC cost. Although the average time and costs of the informal care are 25.0 hours per week and JPY 3.82 million respectively, for those with the highest care need (i.e. nursing care level 5) they are 38.2 hours per week and JPY 6.82 million per year respectively, which is higher than the average annual income in this country [28]. Another important fact is that supervision is not included into consideration at estimating informal care cost in this study. If included, the average informal care time will rise by over 50 hours/ week, reaching close to 70 hours/ week for nursing care level 5. With such a huge burden that can bring on psychological distress to the caregivers, developing social measures to support the caregivers as well as people with dementia is a pressing issue.

The cost of dementia is expected to increase considerably. We estimated that the total cost of dementia will be JPY 24.3 trillion (USD 18.8 billion) in 2060. This means the total cost in 2060 is approximately 1.6 times bigger than that at the moment. However, the total cost per worker by 2060 is expected to be 2.8 times higher than that in 2014 because the size of the labour force population will decrease considerably (i.e. from 76.8 million in 2015 to 44.2 million in 2060 [30]. This result indicates that immediate intervention to mitigate the expected increase of care needs is crucial.

### Limitations

Our estimates of LTC costs may be an overestimate because it was not possible to exclude the costs of LTC due to comorbid diseases. Differentiating the cost attributable to comorbid disease was difficult because the records for receipt of LTC insurance benefits do not include accurate diagnostic data. With regards the estimation of informal care time, in our survey we had to rely on self-report (or reports by the proxy) with regards the diagnosis of dementia. However, because questionnaires were delivered by physicians of people with dementia and by the organizations aiming to assist the caregivers of people with dementia, the risk that people without dementia were included is relatively low. Another limitation relates to the informal care time estimates, for which there may be recall bias.

Attention should be paid to these points when interpreting the results of this study.

## Supporting information

S1 File Supplementary manuscript. (DOCX)

S2 File Supplementary tables. (XLSX)

S1 Dataset Supplementary dataset. (XLSM)
