# Supplementary material for: The estimated cost of dementia in Japan, the most aged society in the world
Source: PLoS One. 2018 Nov 12;13(11):e0206508. doi: 10.1371/journal.pone.0206508 (PMC6231619; doi:10.1371/journal.pone.0206508)
Supplement: S1 File — (DOCX) [file pone.0206508.s001.docx]

Supplementary manuscript

**Title: The Estimated Cost of Dementia in Japan, the most aged society in the world**

Table of contents

1. Estimating healthcare costs 2
2. Estimating long term care (LTC) costs 5
3. Estimating informal care costs 6
4. References 10
5. **Estimating healthcare costs**

Healthcare system in Japan

In Japan, universal healthcare is provided under public health insurance schemes with co-payment between the patients and the health insurance organizations. The reimbursement system is operated by a mixture of two different schemas, fee-for-services and Diagnosis Procedure Combination Per Diem Payment System (DPC/PDPS) (i.e. bundled payment). Under the fee-for-service schema, 30% of the cost relevant to each service is paid out of pocket by the patients and the rest (70% of all the costs) are reimbursed by the health insurance organization to healthcare providers based on the ‘tariff’ for each intervention regulated by the government. All outpatient services are operated on the basis of a fee-for–service schema. With respect to the inpatient services, the mixture of DPC/PDPS and fee-for-service is adopted.

Data sources

As described in the manuscript, we accessed a sampling data set (SDS) randomly extracted from the National Data Base (NDB) (1% for outpatient, 10% for inpatient), a database of all national healthcare insurance receipts. Consequently, the SDS includes 755,247 and 224,509 (i.e. 130,801 for fee-for –service and 93,708 for DPC/PDPS) receipts for outpatient and for inpatient receipts respectively. Each receipt includes information about the patients such as sex, age, all diagnoses, all interventions provided within the healthcare insurance schema, and the total number of days of use of healthcare services at October 2011.

Regression model to estimate the predicted healthcare costs

Using SDS we constructed a regression model to predict healthcare costs. The model was constructed as follows. With respect to inpatient healthcare costs, we used a model with logarithmic transformation because the distribution of inpatient costs appeared not to follow normal distribution.

Although the variable of the total number of days of use of healthcare services might considerable correlations with healthcare cost, we still judged that it should be included as an independent variable due to the reason as follow.

We used the healthcare receipt dataset at October 2011. This means the dataset includes the healthcare service use data only between 1st and 31th October. If a patient was admitted at 15th September and discharged at 1st October (admitted for 17 days), the service use data for just one day (i.e. the cost for 1st October) appears in the dataset, while if he/she was admitted at 1st and discharged at 17^th^ October (admitted for 17 days as well), the data for 17 days would appear. We were afraid that the accuracy to predict the healthcare cost of dementia would decrease if we conducted the analyses without this variable. Actually, in the preliminary analyses, the coefficients of determination of the formulae without this variable were less than 0.01 while those with service days are over 0.4. Therefore we judged it appropriate to keep this variable in the formula.

*HC_dem(inpatient)_ = e^β0^ × e^β1 sex^ × e^β2 age^× e^β3 days^ × e^β4 DEM^ × e^β5 MI^ × e^β6 CHF^ × e^β7 PVD^ × e^β8 CVD^ × e^β9 CPD^ × e^β10 RD^ × e^β11 PUD^ × e^β12 MLD^ × e^β13 DwoC^ × e^β14 DWCC^ × e^β15 HP^ × e^β16 Ren^ × e^β17 MAL^ × e^β18 MSLD^ × e^β19 MST^ × e^β20 AIDS^* + *ε*

*HC_dem(outpatient)_ =β_0_+β_1_sex+β_2_age+β_3_days+β_4_DEM+β_5_MI+β_6_CHF+β_7_PVD+ β_8_CVD+β_9_CPD+β_10_RD+β_11_PUD+β_12_MLD+β_13_DwoC+β_14_DWCC+β_15_HP+β_16_Ren+β_17_MAL+β_18_MSLD+β_19_MST+β_20_AIDS* + *ε*

Where *HC_dem(inpatient/outpatient)_* represents healthcare cost (inpatient/ outpatient) attributable to dementia, *age* is the age of the patient with dementia, *sex* is sex of the patients, *days* is the total number of days healthcare service is provided. Diagnoses are categorized according to Charlson comorbidity index as mentioned below. *e^β0^* represents a constant, while ε represents the residual.

*abbreviation for Charlson comorbidity index^1^

MI: Myocardial infarction, CHF: Congestive heart failure, PVD: Peripheral vascular disease, CVD: Cerebrovascular disease, DEM: Dementia, CPD: Chronic pulmonary disease, RD: Rheumatic disease, PUD: Peptic ulcer disease, MLD: Mild liver disease, DwoC: Diabetes without chronic complication, DWCC: Diabetes with chronic complication, HP: Hemiplegia or paraplegia, RenD: Renal disease, MAL: Any malignancy including lymphoma and leukemia except malignant neoplasm of skin, MSLD: Moderate or severe liver disease, MST: Metastatic solid tumor, AIDS: AIDS/HIV.

The co-efficient and standard error (se) of the parameters are shown in Table S1- S2.

Healthcare costs under DPC/PDPS schema

As previously described, while all outpatient services are reimbursed under fee-for-service scheme, the costs of inpatient services are reimbursed by two different schemas (e.g. fee-for-service and DPC/PDPS). Because information on healthcare costs was not available with respect to the receipts relating to DPC/PDPS, we stratified the healthcare costs under DPC/PDPS by gender and age using the assumption that they were equal to those reimbursed by the fee-for-service schema. To calculate the costs of healthcare under the DPC/PDPS, we applied the unit cost obtained from the fee-for-service scheme, by age and gender, to the population of the same age and gender who use the DPC/PDPS. The percentage of the patients with dementia treated in the schema of fee-for-service and DPC/PDPS are 89% and 11% respectively.

1. **Estimating long term care (LTC) costs**

LTC service system in Japan

In Japan, LTC services are provided under the LTC insurance schema. Those services are provided mainly to the elderly who need support or care to live. People who wish to receive LTC services are assessed to determine the degree of care needed and certified accordingly. The certificates are divided into two categories, need for support and need for nursing care. Patients who are categorized as needing support are likely to have less severe disability, compared to those categorized as needing nursing care. Services under the support category mainly focus on the prevention of disability. On the other hand, those who are categorized as needing nursing care level need a variety of care services to live. There are two levels of “Support”: level 1 and 2, while there are five levels of nursing care. In this paper, we treated these two separate care categories as one categorical variable.

Difference in the cost of users with dementia and without dementia

As mentioned in the manuscript, the average cost of LTC stratified by care needed level was obtained from the result of the Survey of Long-Term Care Benefit Expenditures^2,3^, a nationwide survey on the basis of all individual-level receipts, was comprehensive cost including both that of users with and without dementia. In order to specify the LTC cost of users with dementia, we accessed to the individual receipts for the service provision of the LTC insurance from a local municipality (n=2,245) instead. We evaluated ‘relative ratio’ of the cost between those with and without dementia inside each care needed level by conducting regression analyses with the service costs as dependent variable and age, sex, existence of dementia, and care needed level as independent variables in order to estimate the average costs of the people with dementia in each care needed level by weighing the ‘relative ratio.’

Regression model to estimate ‘relative ratio’ is as follows.

*TC_ltc_ = e^β0^×e^β1x1^×e^β2x2^×e^β3x3^×e^β4x4^* + *ε*

Where *TC_ltc,_,β_0_, x_1_, x_2,_ x_3_, x_4,_ ε* represents the total cost of LTC*,* constant, age, sex, existence of dementia, care needed level, and residual respectively.

The characteristics of the samples and the results of regression analysis depending on service type are shown in Table S3 and S4.

Because the individual receipts of healthcare and LTC are administered separately, it was impossible to combine the data of medical condition (i.e. diagnoses) and service provision of LTC at individual level. Therefore, with respect to LTC cost, it was impossible to exclude the effect of comorbid diseases to LTC cost.

1. **Estimating informal care cost**

Characteristics of the samples

Summary of the characteristics of the samples are shown in Table S5 – S6.

*Care recipients*

Table S5 shows the socio-demographic and clinical characteristics of care recipients. The mean age (standard deviation: sd) of the care recipients of the samples was 83.5 (7.8), and 29.2% were male. With respect to distribution of the care need level, 26.5% of people were in nursing care level 1, followed by nursing care level 2 (22.6%), nursing care level 3 (20.0%), nursing care level 4 (12.7%), nursing care level 5 (9.9%), support need level 2 (4.5%), and support need level 1 (3.8%). The rate of the care recipients who live with other family members was 86.9%. With respect to behavioral and psychological symptoms of dementia (BPSD), delusions (35.6%), visual hallucinations and auditory hallucination (30.2%) were the most common psychiatric symptoms. About the comorbid diseases, hypertension (44.9%) co-existed the most, followed by stroke (19.4%), joint disease, arthritis (19.2%), heart disease (16.3%), diabetes (15.0%), mental disorder (14.2%), cancer (11.8%), and lung disease (7.3%).

*Caregivers*

Table S6 shows socio-demographic characteristics and psychological distress of caregivers. The mean age (sd) of the caregivers was 62.9 (11.7), and 73.3% of them were female. Almost half of caregivers (49.7%) were offspring and 30.0% were spouses of the care recipients respectively. 79.9％ were married and 80.7% lived with the care recipients. Mean number (sd) of caregivers per care recipient was 1.67 (0.91). Mean (sd) of K6 score was 7.79 (5.23).

Constructing a model to predict informal care time

To estimate informal care time of the caregivers in Japan, we constructed a model to predict it by conducting regression analysis using questionnaires samples.

Time spent on informal care was set as a dependent variable, independent variables were sex, age, existence of others living together, total amount of time for LTC service, comorbid diseases (hyper tension, stroke, arthritis, heart disease, diabetes, mental disorders, cancer, respiratory disease, and others), and BPSD such as, hallucination, delusion, day night reversed, verbal abuse, violence, resistance to care, loitering, carelessness with fire, allotriophagy and sexual problem behavior. All independent variables except age, which was set as a continuous variable, were treated as categorical variables.

The regression model was constructed is as follows.

*T_ic_ =β_0_ + β_1_age + β_2_sex +β_3_cnl_1_ + β_4_cnl_2_ + β_5_cnl_3_ + β_6_cnl_4_ + β_7_cnl_5_ + β_8_cnl_6_ + β_9_cnl_7_ + β_10_lt + β_11_hal + β_12_del + β_13_dnr + β_14_abu + β_15_vio + β_16_res + β_17_wan + β_18_cof + β_19_unc + β_20_all + β_21_spb + Β_22_str + β_23_cv + β_24_hp + β_25_pul + β_26_dia + β_27_can + β_28_md + β_29_art* + *ε*

Where *T_ic_* represents informal care time (hrs/week), *β_0_* is constant, *age* is the age of the person with dementia, *sex* is sex of the person with dementia, *cnl_i_* is the care needed level i, *lt* is whether a caregiver lives with a care recipient, *hal* is hallucination, *del* is delusion *dnr* is day-night reversed, *abu* is abusive word, *vio* is violence, *res* is resistance to care, *wan* is wandering, *cof* is carelessness of fire, *unc* is uncleanliness, *all* is allotriophagy, *spb* is sexual problem behavior, *str* is stroke, *cv* is cardiovascular diseases, *hp* is hypertension, *pul* is pulmonary, *dia* is diabetes, *can* is cancer, *md* is mental disorder, *art* is arthritis, while ε represents the residual.

The maximum hours for informal care time per day was set at 18 hours after considering 6 hours for minimum sleep. The results of the regression analysis are shown in Table S7-10.

Estimating informal care time

*Nationally representative parameters*

Because the sample of this survey was not a representative sample in Japan, background characteristics of the sample, such as age, sex, existence of others living together, total amount of time using LTC services, comorbidity, and BPSD might be different from that of representative samples in Japan. Therefore, in order to obtain a more representative estimate the mean and se of amount of time providing informal care, we used nationwide representative data relevant to age, sex, and whether living alone or not and extrapolated into the model.

With respect to the age and sex of the caregivers, we estimated the national distribution from the Survey of Long-term Care Benefit Expenditures ^2,3^. With regards to whether the person with dementia lived alone, because we didn’t obtain any reliable data stratified by nursing care level, we utilized data of the proportion of people aged 65 or more living alone, which we obtained from Household Projections for Japan: 2010-2035^4^ hypothesizing that the rate is applicable even to older people with dementia, irrespective of the care need level. Because we couldn’t find any reliable national data about the total amount of time for formal LTC services, and BPSD, we used relevant data from the samples for extrapolation. To exclude the effect of comorbid diseases to informal care time, we inputed zero into all parameters related to physical comorbid diseases. The parameters imputed in the model, compared to those in samples, are indicated in Table S11.

*Probabilistic analysis*

In order to estimate the mean and se of Japanese representative informal care time stratified by care needed level, we conducted probabilistic analysis for 10,000 times using the model mentioned above, inputing key variables (age, sex, and whether living alone or not), in accordance with mean and se of each parameter. The result is shown in Table 3 in the manuscript.

Estimating expected mean lost wages

In order to estimate the expected mean lost wages, first, we calculated the number of caregivers by sex and age from samples of the survey. Next, the mean wage and labour participation rate by sex and age were drawn from the Basic Survey on Wage Structure ^5^ and Labour Force Survey^6^ and we calculated the expected mean wage by sex and age (the expected mean wage = the mean wage × labour participation rate). Finally, the expected mean wages by sex and age were weighted and averaged by the number of the careers by sex and age of the samples, and was regarded as the expected mean lost wage, being applied to unit cost of IADL in base case analysis.

1. **References**

1. Charlson ME, Pompei P, Ales KL, MacKenzie CR. A new method of classifying prognostic comorbidity in longitudinal studies: development and validation. *Journal of chronic diseases.* 1987;40(5):373-383.

2. Ministry of Health Labour and Welfare of Japan. Survey of Long-Term Care Benefit Expenditures. In: Ministry of Health Labour and Welfare of Japan, ed. Tokyo: Ministry of Health Labour and Welfare of Japan (in Japanese); 2013.

3. Ministry of Health Labour and Welfare of Japan. Survey of Long-Term Care Benefit Expenditures. In: Ministry of Health Labour and Welfare of Japan, ed. Tokyo: Ministry of Health Labour and Welfare of Japan (in Japanese); 2014.

4. Research NIoPaSS. Household Projections for Japan: 2010-2035. 2013; <http://www.ipss.go.jp/pp-ajsetai/j/HPRJ2013/hhprj2013_PRS329.pdf>.

5. Ministry of Health Labour and Welfare of Japan. Basic Survey on Wage Structure (Chingin Kozo Kihon Tokei Chosa). In: Ministry of Health Labour and Welfare of Japan, ed. Tokyo: Ministry of Health Labour and Welfare of Japan (in Japanese); 2014.

6. Ministry of Internal Affairs and Communications of Japan. Labour force survey in 2013. In: Ministry of Internal Affairs and Communications of Japan, ed. Tokyo: Ministry of Internal Affairs and Communications of Japan (in Japanese); 2013.
